# Diversity and Ice Nucleation Activity of Microorganisms Collected With a Small Unmanned Aircraft System (sUAS) in France and the United States

**DOI:** 10.3389/fmicb.2018.01667

**Published:** 2018-08-15

**Authors:** Celia Jimenez-Sanchez, Regina Hanlon, Ken A. Aho, Craig Powers, Cindy E. Morris, David G. Schmale

**Affiliations:** ^1^School of Plant and Environmental Sciences, Virginia Tech, Blacksburg, VA, United States; ^2^Department of Biological Sciences, Idaho State University, Pocatello, ID, United States; ^3^Department of Civil and Environmental Engineering, Virginia Tech, Blacksburg, VA, United States; ^4^INRA, Plant Pathology Research Unit, Provence Alpes Côtes d'Azur Research Center, Montfavet, France

**Keywords:** aerobiology, dispersal, diversity, ice nucleation, UAS, UAV, drone, *Pseudomonas*

## Abstract

Many microbes relevant to crops, domestic animals, and humans are transported over long distances through the atmosphere. Some of these atmospheric microbes catalyze the freezing of water at higher temperatures and facilitate the onset of precipitation. We collected microbes from the lower atmosphere in France and the United States with a small unmanned aircraft system (sUAS). 55 sampling missions were conducted at two locations in France in 2014 (an airfield in Pujaut, and the top of Puy de Dôme), and three locations in the U.S. in 2015 (a farm in Blacksburg, Virginia, and a farm and a lake in Baton Rouge, Louisiana). The sUAS was a fixed-wing electric drone equipped with a remote-operated sampling device that was opened once the aircraft reached the desired sampling altitude (40–50 meters above ground level). Samples were collected on agar media (TSA, R4A, R2A, and CA) with and without the fungicide cycloheximide. Over 4,000 bacterial-like colonies were recovered across the 55 sUAS sampling missions. A positive relationship between sampling time and temperature and concentrations of culturable bacteria was observed for sUAS flights conducted in France, but not for sUAS flights conducted in Louisiana. A droplet freezing assay was used to screen nearly 2,000 colonies for ice nucleation activity, and 15 colonies were ice nucleation active at temperatures warmer than −8°C. Sequences from portions of 16S rDNA were used to identify 503 colonies from 54 flights to the level of genus. Assemblages of bacteria from sUAS flights in France (TSA) and sUAS flights in Louisiana (R4A) showed more similarity within locations than between locations. Bacteria collected with sUAS on TSA in France and Virginia were significantly different across all levels of classification tested (*P* < 0.001 for class, order, family, and genus). Principal Coordinates Analysis showed a strong association between the genera *Curtobacterium, Pantoea*, and *Pseudomonas* from sUAS flights in Virginia, and *Agrococcus, Lysinibacillus*, and *Paenibacillus* from sUAS flights in France. Future work aims to understand the potential origin of the atmospheric microbial assemblages collected with sUAS, and their association with mesoscale atmospheric processes.

## Introduction

Many microorganisms that impact the health of crops, domestic animals, and humans are transported over long distances through the atmosphere (Schmale and Ross, 2015). The dispersal of these microbes in the atmosphere is comprised of a complex series of events (Isard and Gage, 2001). Such events include microbial life history stages (e.g., aerosolization, escape from terrestrial or aquatic environments, survival while airborne, deposition onto a suitable environment, etc.), that interact with biotic and abiotic factors (e.g., rainfall, air currents, UV radiation, etc.) that control dispersal (Isard et al., 2005). These events and factors change in time and space across large-scale biological and meteorological gradients, forming a framework to understand the aerobiological processes governing the spread of microorganisms in the atmosphere (Aylor, 1986; Schmale and Ross, 2015).

Some atmospheric microbes catalyze the freezing of water at higher temperatures and may facilitate the onset of precipitation (Burrows et al., 2009; Murray et al., 2012; Morris et al., 2014). Such ice-nucleating microorganisms likely play an important role in the water cycle (Sands et al., 1982; Morris et al., 2014). Bacteria in the atmosphere may be associated with cloud formation (Ahern et al., 2007; Joly et al., 2013; Hu et al., 2017). A number of studies have described the ice nucleation activity (INA) of microorganisms collected from different components of the water cycle (Burrows et al., 2009; Murray et al., 2012; Morris et al., 2013). It has been hypothesized that the INA phenotype emerged as an adaptation to harsh conditions in the atmosphere; freeze tolerance and/or microbes facilitate their own deposition from the atmosphere (with low nutrient concentration) to land (rich in nutrients) through the production of ice-nucleating proteins or macromolecules (Morris et al., 2014).

An average of 2 × 10^4^ fungal spores and bacteria m^−3^ was reported over many continental regions (Spracklen and Heald, 2014). Residence times of about 3–17 days could be enough to allow the transport of microbes over thousands of kilometers (Mayol et al., 2017). Islands, such as those in the West and Central Pacific, may act as stepping stones for the long-range transport of microorganisms across oceans (Mayol et al., 2017). The modeling of Lagrangian coherent structures (LCS) may help describe the long distance transport of microorganisms within and among habitats (Tallapragada et al., 2011).

New technologies are needed to study the movement of microorganisms in the atmosphere. Previous studies (Schmale et al., 2008, 2012; Lin et al., 2013, 2014) have used unmanned aircraft systems (UASs) to study the transport of microorganisms tens to hundreds of meters above the ground. These UASs are equipped with unique devices for collecting microbes in the atmosphere during flight. Autonomous systems enable teams of UASs to perform complex atmospheric sampling tasks, and coordinate flight missions with one another colleagues (Techy et al., 2010). Data collected with UASs can be used to validate and improve disease forecasting models along highways in the sky, connecting transport scales across farms, states, and continents (Schmale and Ross, 2015). Though terrestrial environments are often considered a major contributor to atmospheric microbial aerosols, little is known about aquatic sources of microbes. Droplets containing microorganisms can aerosolize from the soil and water surface, liberating them into the atmosphere (Pietsch et al., 2015; Joung et al., 2017). Aerosolized droplets may be produced in several ways, including bubble-bursting and fragmentation from breaking waves (Wu, 1981). Fluorescent dyes have been released as surrogate pathogenic microorganisms, and tracked with teams of unmanned systems in the water and in the air (Powers et al., 2018a).

The overall goal of our work was to measure the diversity and ice nucleation activity of microbes in the lower atmosphere. The specific objectives of our work were to: (1) collect microbes from the lower atmosphere at several sites with a small unmanned aircraft system (sUAS), (2) quantify culturable microorganisms from sUAS flights using different types of agar media to maximize the diversity of microorganisms detected, (3) determine the frequency of INA microbes collected with sUAS at −8°C, (4) identify bacteria from sUAS missions using sequences from portions of 16S rDNA, and (5) explore associations of microbial diversity to the different flights (55), locations (Virginia, Louisiana, and France), and media (R4A, TSA, R2A, and CA).

## Materials and methods

GPS locations of field studies are reported below in decimal degrees (DD). Field studies were conducted at two locations in France in 2014; an airfield in Pujaut (43.994737, 4.750301), and the top of Puy de Dôme (45.773260, 2.964211). Field studies were also conducted at three locations in the U.S. in 2015; a farm in Blacksburg, Virginia (37.196049, −80.576872), a farm in Baton Rouge, Louisiana (30.362330, −91.166616), and a lake in Baton Rouge, Louisiana (30.409039, −91.164263) (Table 1). These locations were selected based on their proximity to potential terrestrial and aquatic sources of ice nuclei.

**Table 1:** Flight data for 55 sUAS sampling flights at Pujaut, France; Puy de Dôme, France; Kentland Farm, Blacksburg, Virginia, USA; and Baton Rouge, Louisiana, USA in 2014 and 2015.

**Flight**	**Date (d/m/y)**	**Time**	**Media**	**CFU**	**Microbes/m^3^**	**Altitude**	**Interval (min)**	**Airspeed (km/h)**	**Location**
FRA01	27/03/2014	3:47 pm	TSA + CHX	42	23	~40 m	5	45	Pujaut, France
FRA02	27/03/2014	4:06 pm	TSA + CHX	29	16	~40 m	5	45	Pujaut, France
FRA03	28/03/2014	8:59 am	TSA + CHX	19	5	~40 m	10	45	Pujaut, France
FRA04	28/03/2014	9:41 am	TSA + CHX	46	12	~40 m	10	45	Pujaut, France
FRA05	28/03/2014	10:30 am	TSA + CHX	44	12	~40 m	10	45	Pujaut, France
FRA06	28/03/2014	3:25 pm	TSA + CHX	44	24	~40 m	5	45	Pujaut, France
FRA07	30/03/2014	2:57 pm	TSA + CHX	27	7	~40 m	10	45	Pujaut, France
FRA08	30/03/2014	3:42 pm	TSA + CHX	74	20	~40 m	10	45	Pujaut, France
FRA09	30/03/2014	4:33 pm	TSA + CHX	161	43	~40 m	10	45	Pujaut, France
FRA10	31/03/2014	9:26 am	TSA + CHX	3	1	~40 m	10	45	Pujaut, France
FRA11	31/03/2014	10:11 am	TSA + CHX	15	4	~40 m	10	45	Pujaut, France
FRA12	31/03/2014	11:11 am	TSA + CHX	15	4	~40 m	10	45	Pujaut, France
FRA13	31/03/2014	12:06 pm	TSA + CHX	125	34	~40 m	10	45	Pujaut, France
FRA14	01/04/2014	8:37 am	TSA + CHX	1	0	~40 m	10	45	Pujaut, France
FRA15	01/04/2014	9:40 am	TSA + CHX	6	2	~40 m	10	45	Pujaut, France
FRA16	01/04/2014	10:43 am	TSA + CHX	22	6	~40 m	10	45	Pujaut, France
FRA17	01/04/2014	11:35 am	TSA + CHX	112	30	~40 m	10	45	Pujaut, France
FRA18	01/04/2014	12:40 pm	TSA + CHX	65	17	~40 m	10	45	Pujaut, France
FRA19	01/04/2014	1:33 pm	TSA + CHX	42	11	~40 m	10	45	Pujaut, France
FRA20	04/04/2014	1:03 pm	TSA + CHX	8	3	~30 m	7	45	Puy de Dôme, France
CF21	10/06/2015	9:23 am	R2A	16	4	~40 m	10	45	Kentland Farm, Virginia
CF22	10/06/2015	10:09 am	TSA + CHX	28	8	~40 m	10	45	Kentland Farm, Virginia
CF23	10/06/2015	11:08 am	CA	199	53	~40 m	10	45	Kentland Farm, Virginia
CF24	10/06/2015	11:41 am	R2A + CHX	51	14	~40 m	10	45	Kentland Farm, Virginia
CF25	10/06/2015	12:14 pm	TSA	186	50	~40 m	10	45	Kentland Farm, Virginia
CF26	10/06/2015	1:06 pm	CA + CHX	24	6	~40 m	10	45	Kentland Farm, Virginia
CF27	11/06/2015	9:33 am	CA	39	10	~40 m	10	45	Kentland Farm, Virginia
CF28	11/06/2015	10:13 am	R2A	60	16	~40 m	10	45	Kentland Farm, Virginia
CF29	11/06/2015	10:49 am	TSA	111	30	~40 m	10	45	Kentland Farm, Virginia
CF30	11/06/2015	11:24 am	CA+ CHX	86	23	~40 m	10	45	Kentland Farm, Virginia
CF31	11/06/2015	12:01 pm	R2A + CHX	34	9	~40 m	10	45	Kentland Farm, Virginia
CF32	11/06/2015	12:45 pm	TSA + CHX	16	4	~40 m	10	45	Kentland Farm, Virginia
CF33	12/06/2015	8:57 am	CA	79	21	~40 m	10	45	Kentland Farm, Virginia
CF34	12/06/2015	9:39 am	CA + CHX	7	2	~40 m	10	45	Kentland Farm, Virginia
CF35	12/06/2015	10:19 am	R2A	30	8	~40 m	9:30	45	Kentland Farm, Virginia
CF36	12/06/2015	10:50 am	R2A + CHX	78	33	~40 m	6:15	45	Kentland Farm, Virginia
LA1	28/10/2015	2:15 pm	R4A + CHX	80	12	~50 m	10	60	Ben Hur Farm, Louisiana
LA2	28/10/2015	2:44 pm	R4A	127	19	~50 m	10	60	Ben Hur Farm, Louisiana
LA5	28/10/2015	4:16 pm	R4A + CHX	94	14	~50 m	10	60	Ben Hur Farm, Louisiana
LA6	28/10/2015	4:39 pm	R4A	142	21	~50 m	10	60	Ben Hur Farm, Louisiana
LA7	29/10/2015	11:19 am	R4A + CHX	142	31	~50 m	10	50	BREC Lake, Louisiana
LA8	29/10/2015	11:52 am	R4A	103	22	~50 m	10	50	BREC Lake, Louisiana
LA9	29/10/2015	12:24 pm	R4A + CHX	77	17	~50 m	10	50	BREC Lake, Louisiana
LA10	29/10/2015	12:53 pm	R4A	59	13	~50 m	10	50	BREC Lake, Louisiana
LA11	29/10/2015	5:35 pm	R4A + CHX	58	12	~50 m	10	50	Ben Hur Farm, Louisiana
LA12	29/10/2015	5:52 pm	R4A	230	50	~50 m	10	50	Ben Hur Farm, Louisiana
LA14	30/10/2015	10:06 am	R4A + CHX	144	31	~50 m	10	50	BREC Lake, Louisiana
LA15	30/10/2015	10:34 am	R4A	126	27	~50 m	10	50	BREC Lake, Louisiana
LA16	30/10/2015	11:01 am	R4A + CHX	106	23	~50 m	10	50	BREC Lake, Louisiana
LA17	30/10/2015	11:27 am	R4A	130	28	~50 m	10	50	BREC Lake, Louisiana
LA18	31/10/2015	7:39 am	R4A + CHX	70	15	~50 m	10	50	Ben Hur Farm, Louisiana
LA19	31/10/2015	8:06 am	R4A	368	79	~50 m	10	50	Ben Hur Farm, Louisiana
LA20	31/10/2015	8:33 am	R4A + CHX	15	3	~50 m	10	50	Ben Hur Farm, Louisiana
LA21	31/10/2015	8:58 am	R4A	26	6	~50 m	10	50	Ben Hur Farm, Louisiana
LA22	31/10/2015	9:23 am	R4A + CHX	40	9	~50 m	10	50	Ben Hur Farm, Louisiana

*CFUs per flight were converted to CFU/m^3^ of air sampled as described previously (Aylor et al., 2011; Lin et al., 2014)*.

### Small unmanned aircraft system

**Figure 1:**
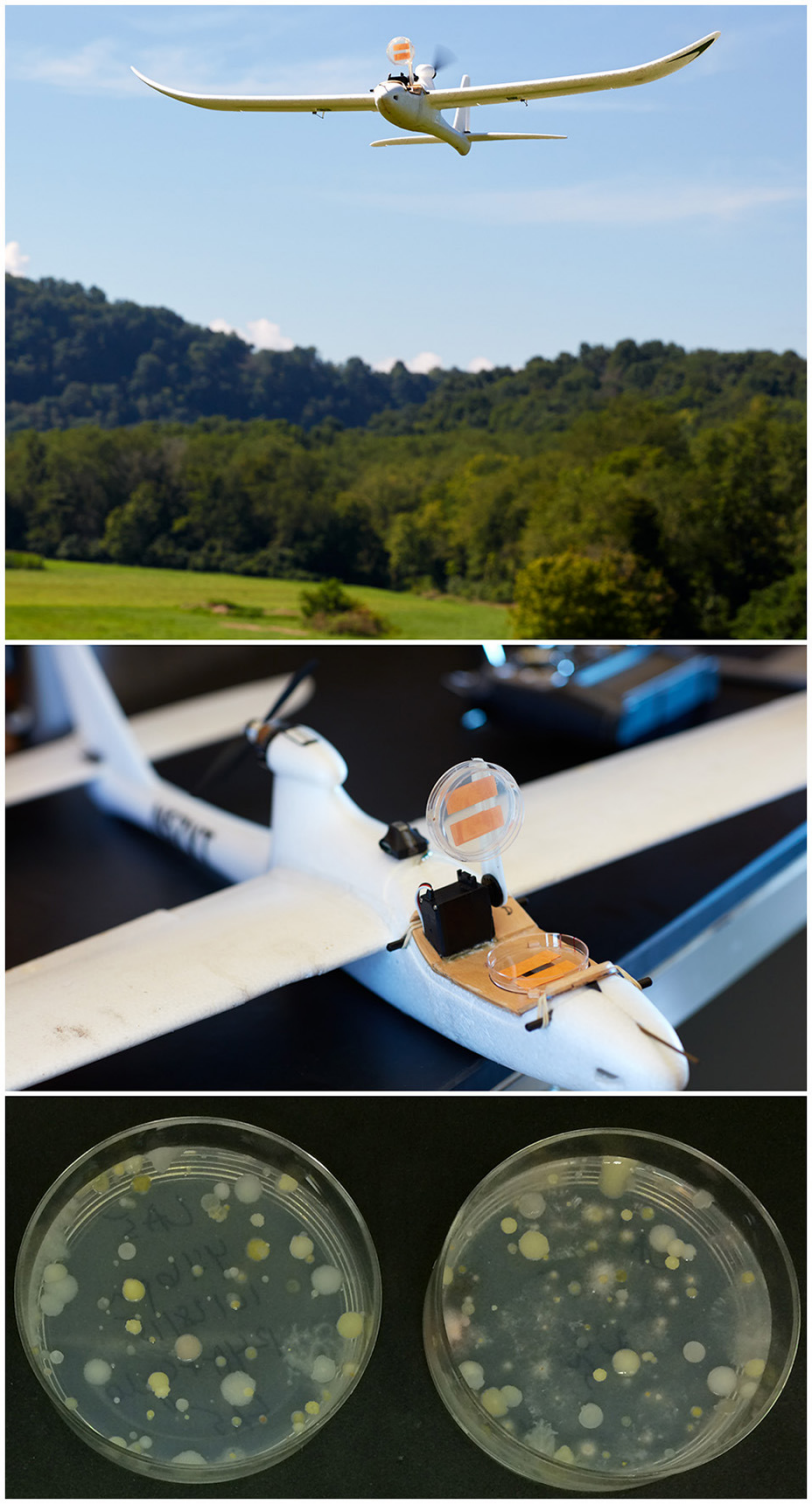
A small unmanned aircraft system (sUAS) was used to collect samples 50 m above ground level. The microbe-sampling device consisted of a single 60 mm vertically-mounted petri plate containing agar media. The sampling device was closed during takeoff and landing, and was opened by remote control from the ground once the sUAS was at the target sampling altitude and airspeed (top and middle panels). The device remained open for the duration of the 10-min sampling interval, and was closed at the end of the sampling interval prior to landing. Immediately following sample collection, the exposed plate containing the agar media was removed from the sampling device and stored in a small plastic container for transport to the laboratory. Plates were incubated for 3–4 days at room temperature, and colony forming units (CFUs) were counted and colonies (bottom panel) were picked and transferred to new media. Left plate was from sUAS mission LA5, and right plate was from sUAS mission LA6.

A small unmanned aircraft system (sUAS) was used to collect microorganisms in the lower atmosphere. The sUAS was an electric fixed-wing drone (the Clouds Fly AXN) with a remote-controlled microbe-sampling device (Figure 1, top). The sUAS was equipped with the Hitec Sensor Station (HTS-SS) including pitot airspeed (HTS-AS) and GPS (HTS-GPS) sensors. The microbe-sampling device consisted of a single 60 mm vertically-mounted petri plate containing agar media (Figure 1, top). This is a modification of the design used by Schmale et al. (2008), Schmale et al. (2012), and Lin et al. (2013, 2014). The sampling device was closed during takeoff and landing and was opened by remote control from the ground once the sUAS was at the target sampling altitude and airspeed. The device remained open for the duration of the sampling interval which was usually 10 minutes, as detailed in Table 1. Immediately following sample collection, the exposed plate containing the agar media was removed from the sampling device and stored in a small plastic container for transport to the laboratory.

### Collection and culturing of microorganisms

Agar collection plates from the sUAS sampling missions contained TSA, R2A, R4A, and CA media with or without 50 mg/L cycloheximide. These media were selected based on their use in previous published reports (Failor et al., 2017; Hanlon et al., 2017), and in an effort to maximize the potential diversity of microorganisms cultured from environmental samples. Plates were incubated for 3–4 days at room temperature, and colony forming units (CFUs) were counted and the plates were photographed (Figure 1, bottom). Colonies were picked with sterile toothpicks and streaked onto the same media they were collected on to obtain pure cultures in a laminar flow hood under sterile working conditions. Pure cultures were stored at −80°C in 20% glycerol.

Individual microbes from France flights were inoculated onto TSA stabs in 1.5 mL Eppendorf tubes for transport back to the laboratories at Virginia Tech, USA. The French collection was cultured on fresh TSA plates prior to INA screening. Flight plates from the LSU and Blacksburg locations were incubated at room temperature, approximately 22°C, for 4–7 days and then stored at 4°C. Microbes were moved as individual isolates to fresh streak plates, or chosen directly from flight plates for INA screening. Samples were then picked as individual microbes, from streak plates or flight plates, and inoculated directly into the 96-well plate for droplet freezing assays.

### Weather data

Historical hourly weather data for each of the regions where the sUAS sampling was conducted were obtained from https://www.wunderground.com/ (Table 2). For Louisiana, weather data were obtained from the Baton Rouge Metro-Ryan airport (https://www.wunderground.com/history/airport/KBTR/). For Virginia, weather data were obtained from the Virginia Tech airport (https://www.wunderground.com/history/airport/KBCB/). For Pujaut, France weather data were obtained from the Caumont airport (https://www.wunderground.com/history/airport/LFMV/). For Puy de Dôme, France weather data were obtained from the Clermont Ferrand airport (https://www.wunderground.com/history/airport/LFLC/).

**Table 2:** Historical weather data for the 55 sUAS flights in France, Virginia, and Louisiana.

**Flight**	**Local time of data**	**Temp**.	**Dew point**	**Humidity(%)**	**Pressure**	**Visibility**	**Wind dir**	**Wind speed**	**Precip**	**Conditions**
FRA01	3:30 p.m.	62.6°F	32.0°F	32	29.74 in	6.2 mi	NNW	10.4 mph	N/A	Unknown
FRA02	4:00 p.m.	62.6°F	32.0°F	32	29.74 in	6.2 mi	NNW	12.7 mph	N/A	Unknown
FRA03	9:00 a.m.	48.2°F	35.6°F	62	29.98 in	6.2 mi	Variable	3.5 mph	N/A	Unknown
FRA04	9:30 a.m.	51.8°F	37.4°F	58	29.98 in	6.2 mi	Variable	3.5 mph	N/A	Unknown
FRA05	10:30 a.m.	59.0°F	39.2°F	48	29.98 in	6.2 mi	SSE	9.2 mph	N/A	Unknown
FRA06	3:30 p.m.	64.4°F	39.2°F	40	29.98 in	6.2 mi	SSW	10.4 mph	N/A	Unknown
FRA07	3:00 p.m.	64.4°F	48.2°F	56	30.01 in	6.2 mi	West	5.8 mph	N/A	Very cloudy
FRA08	3:30 p.m.	64.4°F	48.2°F	56	29.98 in	6.2 mi	West	6.9 mph	N/A	Partly cloudy
FRA09	4:30 p.m.	66.2°F	50.0°F	56	29.98 in	6.2 mi	West	9.2 mph	N/A	Very cloudy
FRA10	9:30 a.m.	50.0°F	44.6°F	82	30.04 in	6.2 mi	Variable	3.5 mph	N/A	Unknown
FRA11	10:00 a.m.	55.4°F	46.4°F	72	30.04 in	6.2 mi	Variable	2.3 mph	N/A	Unknown
FRA12	11:00 a.m.	59.0°F	44.6°F	59	30.04 in	6.2 mi	Variable	2.3 mph	N/A	Unknown
FRA13	12:00 p.m.	64.4°F	44.6°F	49	30.04 in	6.2 mi	Variable	1.2 mph	N/A	Unknown
FRA14	8:30 a.m.	50.0°F	46.4°F	87	29.98 in	6.2 mi	SSE	6.9 mph	N/A	Very cloudy
FRA15	9:30 a.m.	51.8°F	46.4°F	82	30.01 in	6.2 mi	SE	3.5 mph	N/A	Cloudy
FRA16	10:30 a.m.	55.4°F	48.2°F	77	30.01 in	6.2 mi	SSE	6.9 mph	N/A	Cloudy
FRA17	11:30 a.m.	59.0°F	48.2°F	67	30.01 in	6.2 mi	SSE	3.5 mph	N/A	Cloudy
FRA18	12:30 p.m.	60.8°F	50.0°F	68	30.01 in	6.2 mi	SSE	6.9 mph	N/A	Cloudy
FRA19	1:30 p.m.	60.8°F	50.0°F	68	30.01 in	6.2 mi	South	8.1 mph	N/A	Cloudy
FRA20	1:00 p.m.	57.2°F	46.4°F	67	29.80 in	6.2 mi	Variable	2.3 mph	N/A	Cloudy
CF21	9:15 a.m.	66.4°F	60.8°F	82	30.06 in	7.0 mi	No wind	No wind	N/A	Clear
CF22	9:55 a.m.	69.1°F	61.0°F	75	30.06 in	10.0 mi	No wind	No wind	N/A	Clear
CF23	11:15 a.m.	74.8°F	61.2°F	62	30.04 in	10.0 mi	No wind	No wind	N/A	Clear
CF24	11:35 a.m.	75.9°F	61.5°F	61	30.04 in	10.0 mi	No wind	No wind	N/A	Clear
CF25	12:15 p.m.	76.5°F	58.6°F	54	30.04 in	10.0 mi	No wind	No wind	N/A	Clear
CF26	12:55 p.m.	77.9°F	59.2°F	52	30.03 in	10.0 mi	No wind	No wind	N/A	Clear
CF27	9:35 a.m.	68.4°F	65.5°F	90	30.12 in	4.0 mi	No wind	No wind	N/A	Scattered clouds
CF28	10:15 a.m.	72.0°F	66.0°F	81	30.12 in	7.0 mi	NNW	3.5 mph	N/A	Clear
CF29	10:55 a.m.	75.9°F	65.1°F	69	30.13 in	10.0 mi	No wind	No wind	N/A	Clear
CF30	11:15 a.m.	77.5°F	66.0°F	68	30.13 in	10.0 mi	No wind	No wind	N/A	Clear
CF31	12:15 p.m.	80.8°F	65.5°F	59	30.11 in	10.0 mi	WSW	3.5 mph	N/A	Clear
CF32	12:55 p.m.	81.9°F	63.7°F	54	30.10 in	10.0 mi	No wind	No wind	N/A	Clear
CF33	8:55 a.m.	72.0°F	66.6°F	83	30.14 in	10.0 mi	West	3.5 mph	0.03 in	Clear
CF34	9:35 a.m.	74.7°F	66.9°F	77	30.14 in	10.0 mi	West	3.5 mph	0.15 in	Scattered clouds
CF35	10:15 a.m.	76.1°F	66.7°F	73	30.14 in	10.0 mi	West	5.8 mph	N/A	Clear
CF36	10:55 a.m.	77.5°F	66.6°F	69	30.13 in	10.0 mi	West	3.5 mph	0.58 in	Scattered clouds
LA1	1:53 p.m.	80.1°F	62.1°F	54	29.83 in	10.0 mi	WSW	11.5 mph	N/A	Partly cloudy
LA2	2:53 p.m.	81.0°F	62.1°F	52	29.82 in	10.0 mi	WSW	13.8 mph	N/A	Scattered clouds
LA5	3:53 p.m.	82.0°F	62.1°F	51	29.80 in	10.0 mi	SSW	10.4 mph	N/A	Clear
LA6	4:53 p.m.	81.0°F	62.1°F	52	29.78 in	10.0 mi	SSW	13.8 mph	N/A	Partly cloudy
LA7	10:53 a.m.	73.0°F	63.0°F	71	29.97 in	10.0 mi	Variable	4.6 mph	N/A	Partly cloudy
LA8	11:53 a.m.	75.9°F	63.0°F	64	29.97 in	10.0 mi	Variable	5.8 mph	N/A	Clear
LA9	11:53 a.m.	75.9°F	63.0°F	64	29.97 in	10.0 mi	Variable	5.8 mph	N/A	Clear
LA10	12:53 p.m.	77.0°F	62.1°F	60	29.96 in	10.0 mi	Variable	4.6 mph	N/A	Clear
LA11	4:53 p.m.	79.0°F	55.9°F	45	29.91 in	10.0 mi	WSW	4.6 mph	N/A	Clear
LA12	5:53 p.m.	75.0°F	57.0°F	53	29.91 in	10.0 mi	WNW	4.6 mph	N/A	Clear
LA14	9:53 a.m.	69.1°F	60.1°F	73	30.03 in	10.0 mi	East	11.5 mph	N/A	Clear
LA15	10:53 a.m.	73.9°F	62.1°F	66	30.03 in	10.0 mi	East	9.2 mph	N/A	Clear
LA16	10:53 a.m.	73.9°F	62.1°F	66	30.03 in	10.0 mi	East	9.2 mph	N/A	Clear
LA17	11:53 a.m.	78.1°F	61.0°F	56	30.01 in	10.0 mi	ESE	12.7 mph	N/A	Scattered clouds
LA18	7:37 a.m.	73.0°F	68.0°F	84	29.83 in	10.0 mi	ESE	8.1 mph	N/A	Cloudy
LA19	8:00 a.m.	73.0°F	69.1°F	87	29.83 in	9.0 mi	ESE	11.5 mph	-	Light rain
LA20	8:53 a.m.	73.0°F	70.0°F	90	29.83 in	10.0 mi	ESE	10.4 mph	N/A	Cloudy
LA21	8:53 a.m.	73.0°F	70.0°F	90	29.83 in	10.0 mi	ESE	10.4 mph	N/A	Cloudy
LA22	9:53 a.m.	75.0°F	70.0°F	84	29.81 in	10.0 mi	SE	15.0 mph	N/A	Very cloudy

Associations of concentrations of culturable bacteria (CFUs/m^3^) with each of the weather variables for sUAS flights in France and Louisiana (where the same media was used across each of the flights at each location) were examined using regression analysis.

### Ice nucleation assays

Ice nucleation assays were performed in 96-well plates. Pure cultures of microbes were transferred from culture plates with a sterile toothpick to 140 μL of DI water. Plates were covered with pierceable foil (LMT-ALUMA-II, Phenix Research Products Inc, Candler, NC) to prevent cross-contamination across wells and incubated at 4°C for 1 h. The foil was pierced with a multichannel pipet to load the cooling bath, and a second pierceable foil was placed on top after 12 μL sample aliquots were loaded. Droplet freezing assays were performed on a PARAFILM® M (Sigma P6543, 20 in. × 50 ft) float assembly in a Lauda Alpha RA 12 (LCKD 4908) cooling bath (LAUDA-Brinkmann, LP, Delran, NJ, 08075) with ethylene glycol coolant fluid (Air gas RAD64000246). Droplets (12 μL) of the microbial suspension were loaded in duplicate, and the temperature of the cooling bath was decreased stepwise every 2 minutes from −2° to −12°C, or until frost spontaneously formed on the float. Microbes were considered to be INA if at least one drop froze at or before −8°C. These putative INA strains identified from the original test were grown individually on their respective media type and stored in 20% glycerol at −80°C. These isolates were plated from frozen glycerol stocks to test for INA a second time (Table 3).

**Table 3:** Ice nucleation activity (INA) detected in the collection by a droplet freezing assay.

**Location**	**Flight #**	**# Isolates transferred from sUAS collection**	**# INA (orig)**	**% INA (orig)**	**# INA (rep)**	**% INA (rep)**
Louisiana	LA1	64	6	0.09	0	0
Louisiana	LA2	64	2	0.03	0	0
Louisiana	LA6	64	1	0.02	1	0.02
Louisiana	LA7	64	1	0.02	0	0
Virginia	CF30	40	1	0.03	0	0
Virginia	CF32	16	2	0.13	0	0
Virginia	CF35	30	2	0.07	0	0

*A total of 1,963 isolates was screened, and in the first (orig) test, 15 (0.76%) isolates froze droplets at −8°C. Putative ice nucleators were screened again using the same droplet freezing method, and only 1 isolate from Flight LA6 was INA after the second test*.

### Identification of microorganisms using portions of 16S rDNA

All cultures (regardless of INA phenotype) from cryogenic storage were streaked on R2A plates to ensure isolates were pure prior to sequencing. When a mix of different colonies was isolated from a cryogenic sample, additional streaks were performed to ensure the purity of each individual sample. Up to 15 isolates per flight were randomly selected (using assignments based on a random number generator) from each flight collection for sequence-assisted identification.

Colony PCR was used to amplify a portion of 16S rDNA. Different colony PCR strategies were performed on the cultures including: (1) transferring the colony directly to a PCR tube to serve as a template, (2) transferring a tooth-pick microbial biomass to 100 μL of distilled water, and then using 5 μL of this dilution as the template for PCR, (3) heating 10 μL of the cited biomass solution and taking 2 μL as the template, and (4) using a DNA extraction kit (Puregene Yeast/Bact. Kit B; Qiagen #1042607) with microbial glycerol stock as the template for PCR.

A portion of the 16S rDNA gene was amplified using the primers 518 F (5′ CCA GCA GCC GCG GTA ATA CG 3′) and 1491R (5′ ATC GGY TAC CTT GTT ACG ACT TC 3′) (Turner et al., 1999) and http://www.earthmicrobiome.org/empstandard-protocols/16s/). GoTaq® Green Master Mix (Promega M712) was used with 2–5 μL of template in a 20 μL PCR reaction. The parameters used in the thermal cycler were: 1 × cycle denaturation 95°C for 10 min, 5 × cycle denaturation 95°C for 1 min, annealing 53°C for 30 s, extension 72°C for 1min 40 s, 25 × cycle 94°C for 30 s, 53°C for 20 s, 72°C for 1 min, 1 × cycle 72°C for 10 min, hold 4°C). Resulting PCR products were loaded on an 1% TBE agarose gel with Ethidium bromide and visualized on a UV transilluminator. Enzymes rSAP and ExoI were used to clean-up the PCR products, and the cleaned products were sequenced by Eton Biosciences (AB1 3,730 × 1 DNA Sequencer, Eton Biosciences, 104 T.W. Alexander Drive, Bldg 4A, RTP, NC 27709, USA). The 4 Peaks program (Version 1.8. The Netherlands) was used to trim the resulting sequences with a standard quality (QC) of 30 or higher. The trimmed sequences then were queried against the NCBI nr (non-redundant) database to identify the microorganisms to the level of genus. Sequences were also tested to find chimeras using the DECIPHER program, Version 2.0 (Wright et al., 2012). Accession numbers were obtained from GenBank following submission using the BankIt submission tool at NCBI (ncbi.nlm.nih.gov).

### Analysis of microbial diversity among sUAS collections

Identities from sequences from portions of 16S rDNA were used to associate each isolate with class, order, family, and genus. Media effects (CA, R2A, TSA) were considered for flights conducted in Virginia, since this was the only location for which three media types were used. Location effects were ascertained by comparing isolates from TSA media for France and Virginia. Null hypothesis tests of identical assemblage composition among treatments (i.e., media types and location) were run using PERMANOVA (Anderson, 2001) for the taxonomic levels of class, order, family, and genus using 10,000 permutations. Patterns of genera composition were graphically summarized using PCoA (Principal Coordinates Analysis, i.e., Metric multidimensional scaling) (Kruskal, 1964a) (Kruskal, 1964b). To account for presence/absence nature of data, Jaccard's presence/absence index (Jarccard, 1908) was used to create required dissimilarity matrices for analyses. Vector fitting (Oksanen, 2015) was used to depict the strength of association between the ordination projection and the genera that underlay the projection. Vector fitting tests for the null hypothesis of no association were implemented using 1,000 permutations.

## Results

Fifty-five sampling missions were conducted at two locations in France in 2014, and three locations in the U.S. in 2015. Over 4,000 colonies were recovered across the 55 sUAS sampling missions (Table 1).

### Concentrations of microbes

**Figure 2:**
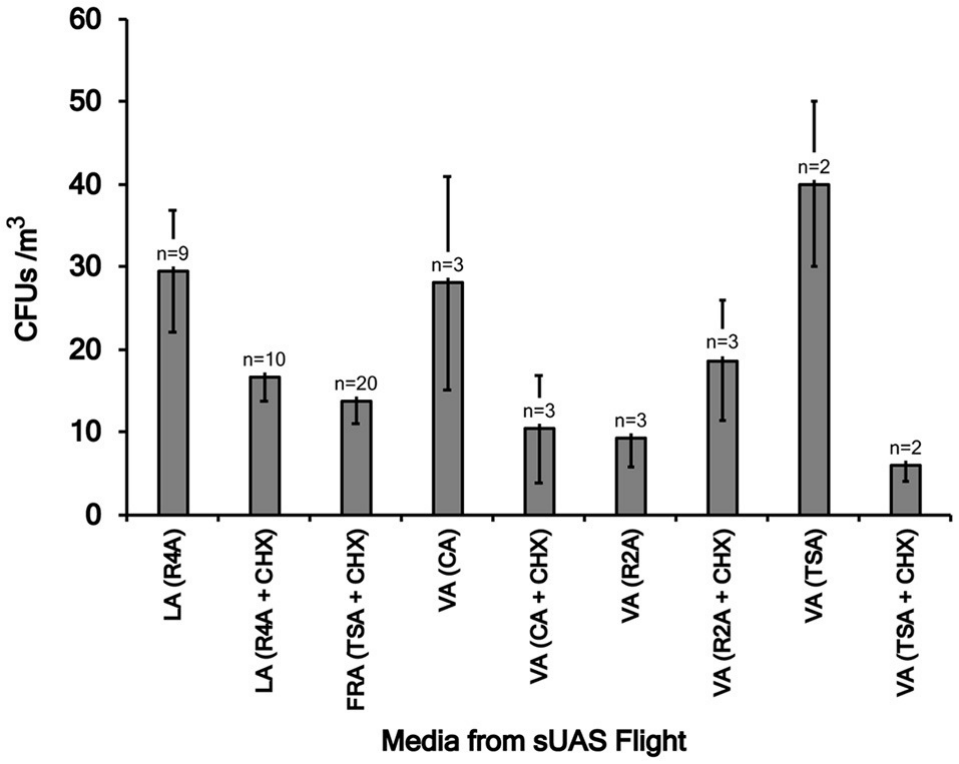
Mean colony forming units (CFUs) on eight different types of media (R4A, TSA, R2A, and CA, with and without 50 mg/L cycloheximide, CHX) for sUAS flights conducted in Louisiana (LA), France (FRA), and Virginia (VA). Mean CFUs were determined for a group of flights in the same location and where the samples were collected on the same media type. The lowest CFU value was 22 taken at Virginia on TSA + CHX media. The highest CFU/mL value were 148 and 146, taken at Virginia (on TSA media) and at Louisiana (on R4A media), respectively.

CFUs per flight were converted to CFU/m^3^ of air sampled as described previously (Aylor et al., 2011; Lin et al., 2014). For flights conducted in Virginia, concentrations of microbes ranged from 2 to 53 (Flight CF23) CFUs/m^3^ (Table 1, Figure 2). For flights conducted in Louisiana, concentrations of microbes ranged from 3 to 79 (Flight LA19) CFUs/m^3^ (Table 1, Figure 2). For flights conducted in France, CFUs ranged from 0 to 43 (Flight FRA09) CFUs/m^3^ (Table 1, Figure 2). Concentrations of microbes were generally lower for media containing the fungicide cylohexamide (CHX) (Figure 2).

**Figure 3:**
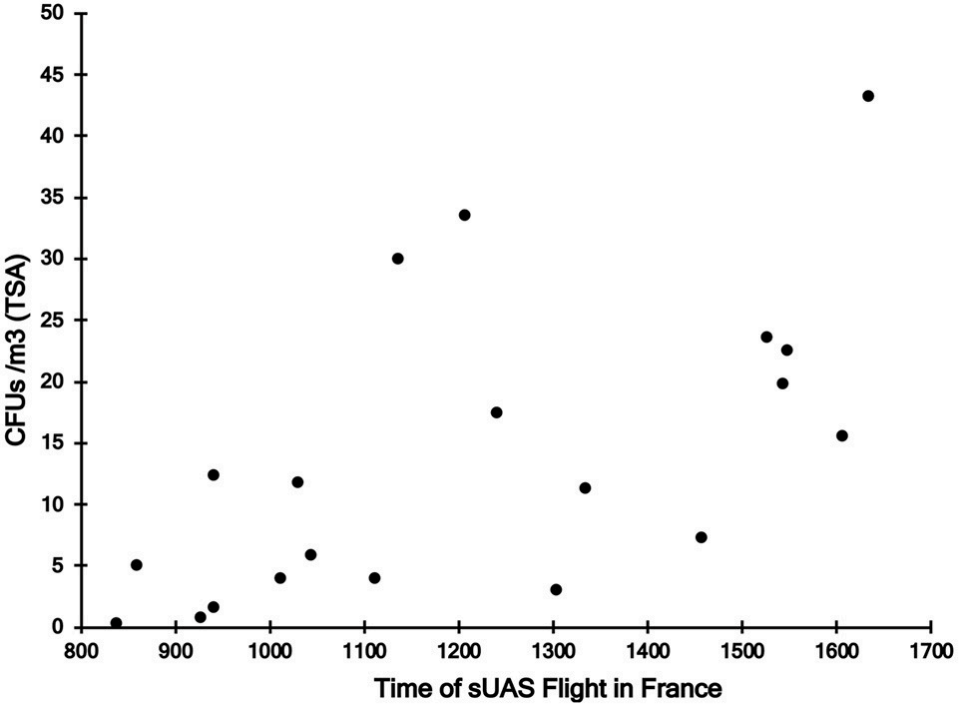
Relationship between sampling time and concentrations of culturable bacteria on TSA (CFU/m^3^) for sUAS flights conducted in France.

For sUAS flights in France, there was a significant positive relationship between time of the sUAS flight (*r* = 0.61, *P* < 0.01) (Figure 3) and temperature during the sUAS flight (*r* = 0.71, *P* < 0.01) and CFU/m^3^. There was a significant negative relationship between relative humidity during the sUAS flight and CFU/m^3^ (*r* = 0.52, *P* = 0.02). There was also a significant negative relationship between relative humidity and temperature (*r* = 0.67, *P* < 0.01). There was no significant relationship between windspeed and CFU/m^3^ (*r* = 0.27, *P* = 0.26).

For sUAS flights in Louisiana, there was no significant relationship between any of the variables tested (time of the sUAS flight, temperature, relative humidity, and windspeed) and concentrations of viable bacteria (CFU/m^3^) (*P* > 0.05 for all variables tested).

### INA strains

The ice nucleation activity (INA) of the isolates collected from the sUAS flights was determined using a droplet freezing assay. A total of 1,963 isolates was tested for INA, and 15 isolates (~1% of the total screened) were INA at −8°C following the initial screen. However, only one of these isolates (LA6P5) showed INA following a second screen from cryogenic cultures. This isolate (LA6P5) was identified as *Xanthomonas* (GenBank accession number MG924929) (Table 4). None of the control droplets containing DI water froze at −12°C (the lower temperature limit) in any of the assays.

**Table 4:** Information on strains collected with sUAS that showed INA at −8°C after the initial screen.

**Flight**	**Strain ID**	**Genus**	**GenBank accesion number**
CF30	CF30P39	*Pantoea*	MG924921
CF32	CF32P4	*Curtobacterium*	MG860096
CF32	CF32P6	*Pseudomonas*	MH014870
CF35	CF35P26	*Arthrobacter*	MG924918
CF35	CF35P27	*Arthrobacter*	MG924919
LA1	LA1P5B	*Curtobacterium*	MG924922
LA1	LA1P6	*Bacillus*	MG924923
LA1	LA1P7	*Acinetobacter*	MG860145
LA1	LA1P10A	*Bacillus*	MG924924
LA1	LA1P10B	*Massilia*	MG924925
LA1	LA1P61	*Bacillus*	MG924926
LA2	LA2P4	*Pseudomonas*	MG924927
LA2	LA2P11	*Bacillus*	MG924928
LA6	LA6P5	*Xanthomonas*	MG924929
LA7	LA7P61	*Bacillus*	MG924930

*Strain LA6P5 showed INA following a second screen from cryogenic cultures*.

### Diversity of microbes in sUAS collections

**Figure 4:**
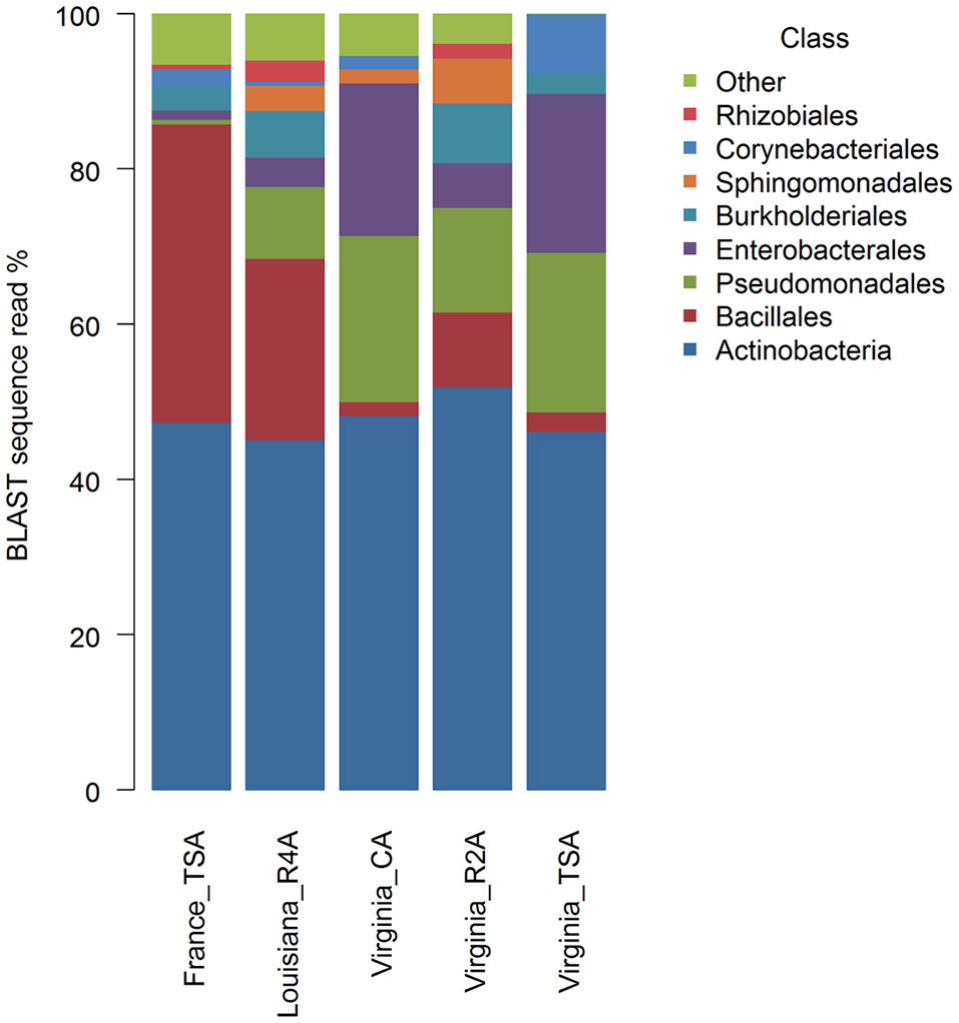
Graphical summaries of 16S taxa in location/media combinations for the level of class.

**Figure 5:**
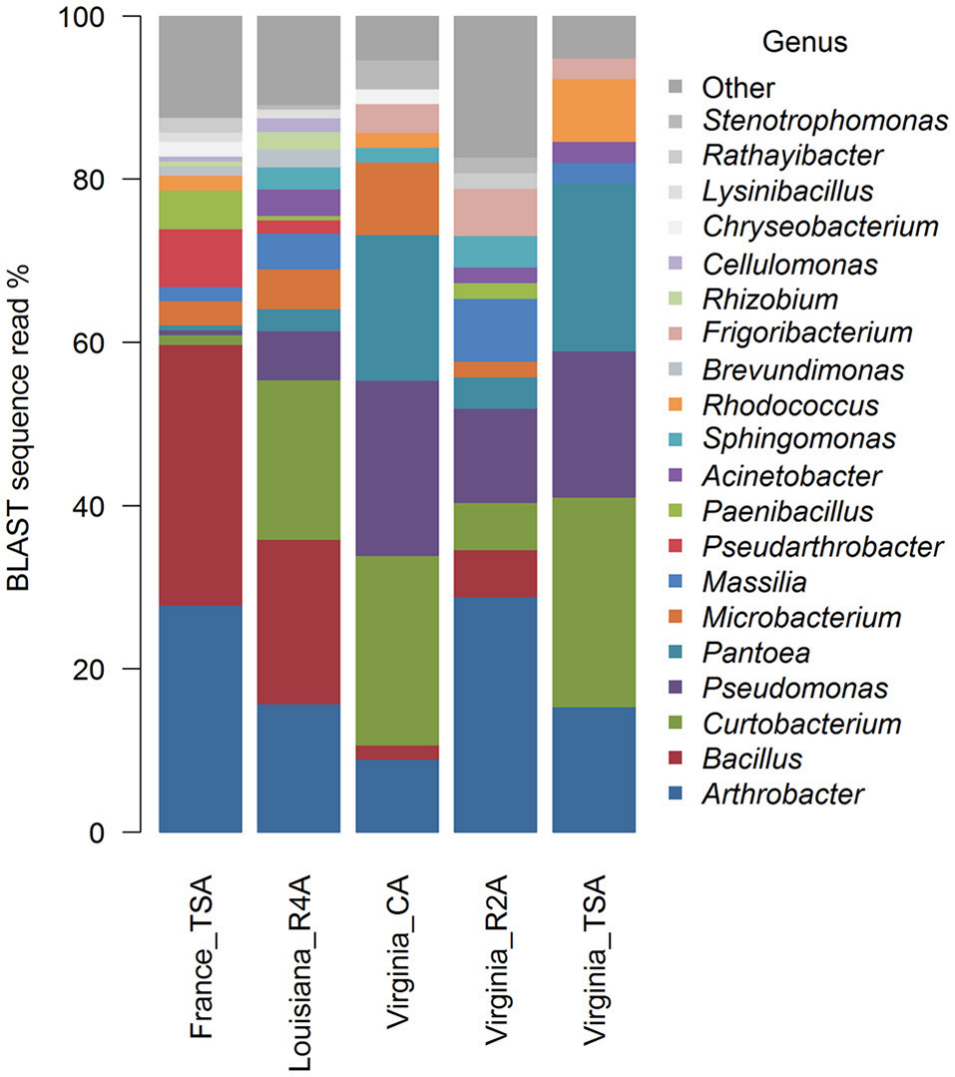
Graphical summaries of 16S taxa in location/media for the level of genus.

Graphical summaries of taxa in location/media combinations are given for class (Figure 4) and for genus (Figure 5). Analyses on the effect of media for sUAS flights in Virginia showed evidence of assemblage variation at the level of genus (*P* = 0.05). No evidence of assemblage variation with media was observed at the levels of class (*P* = 0.34), order (*P* = 0.34), and family (*P* = 0.12). Three genera were significantly associated with projection scatter. These were: (1) *Curtobacterium*, which was associated with CA and TSA (which showed tight clustering), (2) *Microbacterium* which was associated with three of the CA samples, and (3) *Massilia* which was associated with three of the R2A samples.

**Figure 6:**
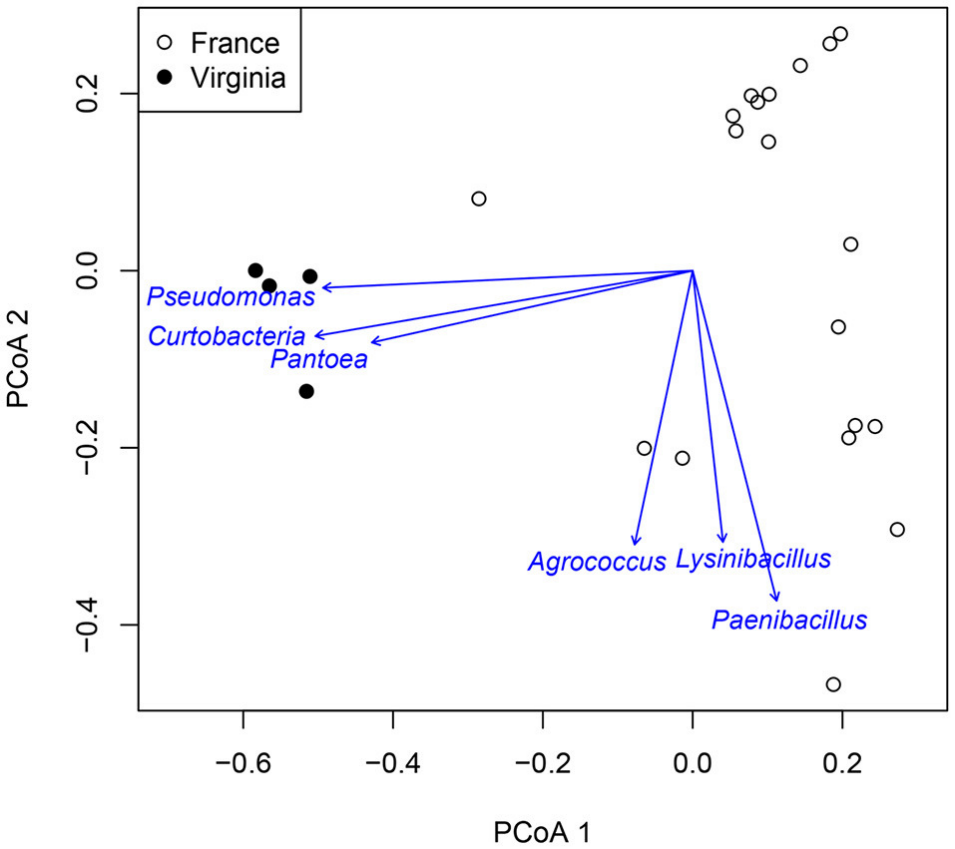
PCoA of Virginia and France genus data for TSA media. Arrows point to the region of most rapid increase in the projection. Arrow length is scaled by strength of correlation to the projection.

Atmospheric assemblages of bacteria collected with sUAS on TSA in France and Virginia were significantly different across all levels of classification tested (*P* < 0.001 for class, order, family, and genus). Principal Coordinates Analysis showed a strong association between the genera *Curtobacterium, Pantoea*, and *Pseudomonas* from sUAS flights in Virginia, and *Agrococcus, Lysinibacillus*, and *Paenibacillus* from sUAS flights in France (Figure 6).

**Figure 7:**
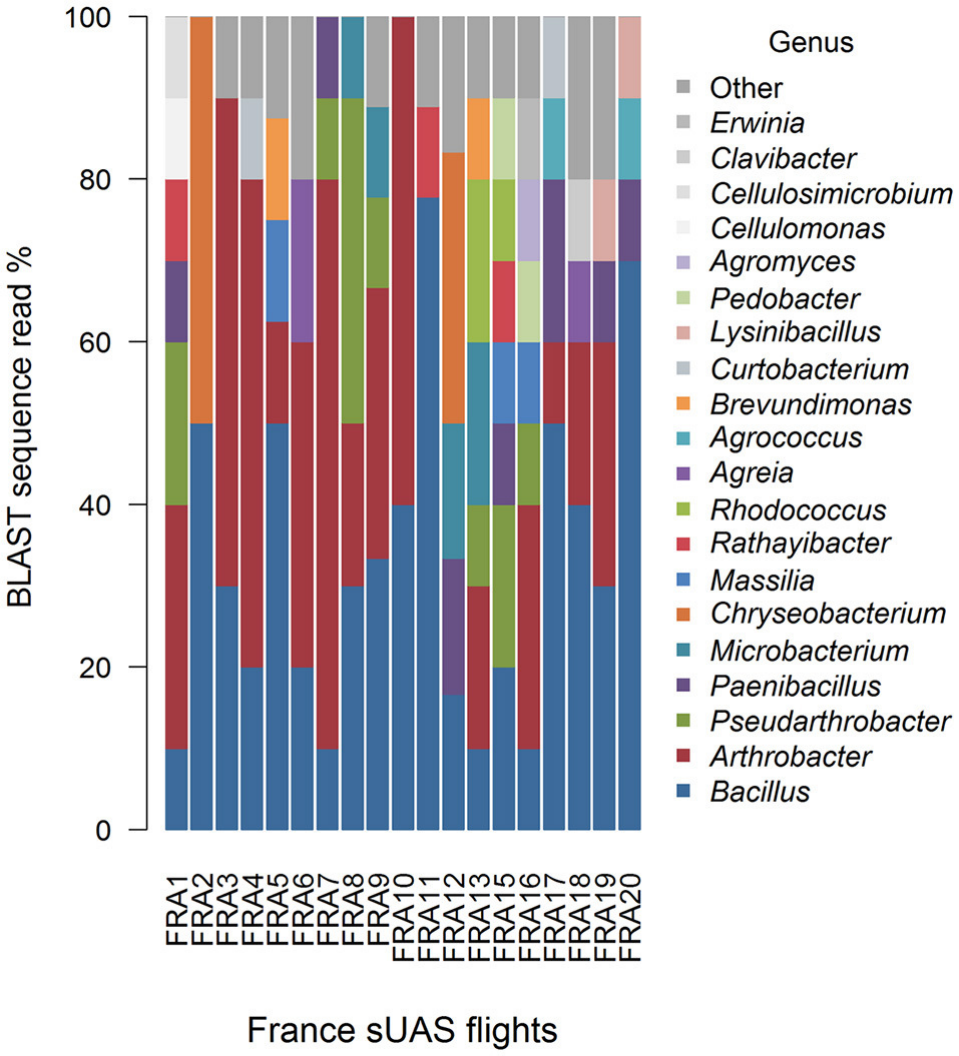
Graphical summaries of 16S taxa for the level of genus for sUAS flights from France on TSA media.

**Figure 8:**
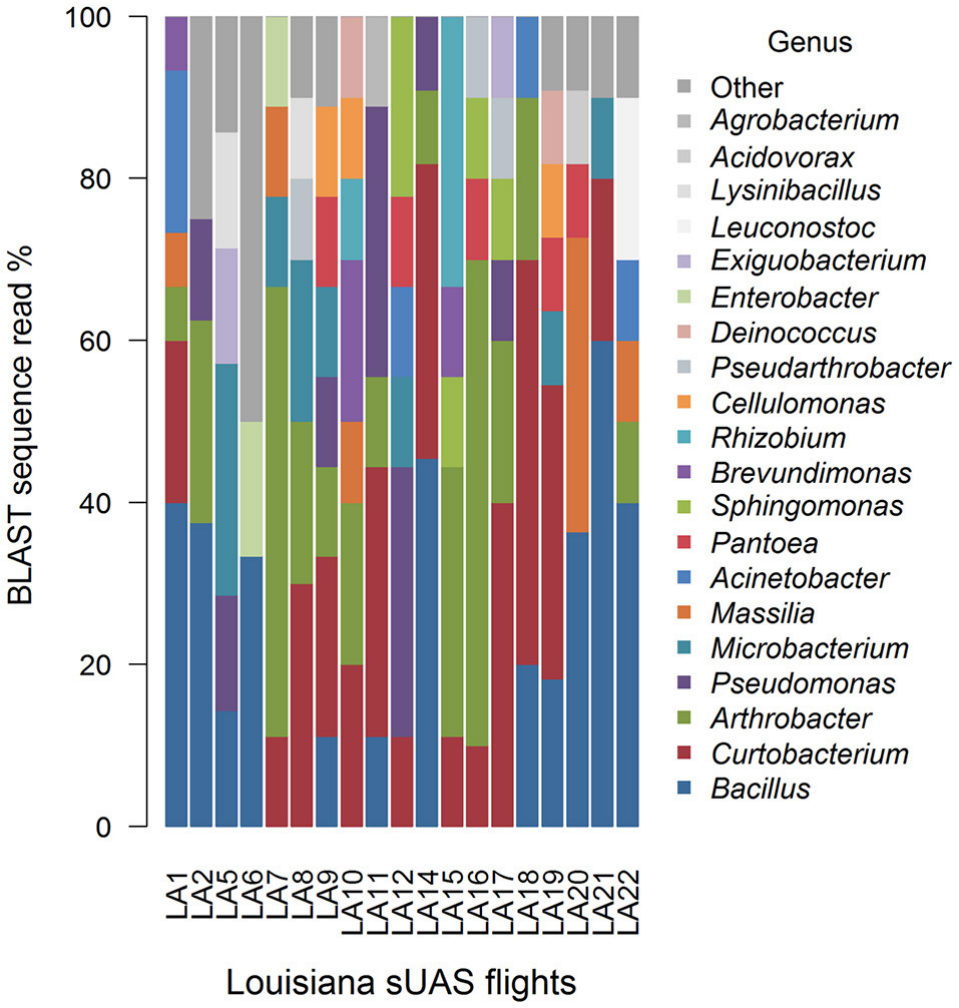
Graphical summaries of 16S taxa for the level of genus for sUAS flights from Louisiana on R4A media.

Mean Jaccard genus-based dissimilarities of sUAS flights in France (TSA) (Figure 7) and sUAS flights in Louisiana (R4A) (Figure 8) showed more similarity within locations than between locations; 0.74 for France|France and 0.78 for Louisiana|Louisiana, and 0.82 for France|Louisiana (Koleff et al., 2003). This is also supported with PERMANOVA testing of the H_0_ that there are no true community differences between France (TSA media) and Louisiana (R4A media) (*P* < 0.0001).

## Discussion

Little is known about the transport, diversity, and ice nucleation activity of microorganisms in the lower atmosphere. We collected microbes from the lower atmosphere in France and the United States with a small unmanned aircraft system (sUAS). At least one culturable microorganism was recovered from all of the 55 sUAS sampling missions, with concentrations of culturable microbes up to 79 CFUs per cubic meter of air sampled. Observed concentrations from our sUAS missions are similar to previous reports in the literature; for example, aerial sampling missions conducted during a dust storm in Boston between April and November 1934 reported between 16 and 266 bacteria/m^3^ of air (Proctor, 1935). Mayol et al. (Mayol et al., 2017) reported airborne concentrations of about 8,000 per m^3^ over the North Atlantic Ocean.

Over 4,000 CFUs were recovered across the 55 sUAS sampling missions on four different types of agar media (TSA, R4A, R2A, and CA) with and without the fungicide cycloheximide. Schmale and colleagues have reported collections of *Fusarium* from the atmosphere using large UAS (Tallapragada et al., 2011; Schmale et al., 2012; Lin et al., 2013, 2014), but most of these collections used a selective medium (a modified Nash-Snyder medium containing the fungicide PCNB and a cocktail of antibiotics) which biased the recovery of fungi in this genus. To our knowledge, our work represents the first published report of microbial exploration in the lower atmosphere using a hand-launched drone. Since our sUAS can be launched by hand, this work demonstrates that such sUAS can be deployed in remote locations lacking an established runway for takeoff and landing (a requirement for large fixed wing drones), thereby opening new perspectives for exploration of tropospheric aerobiology and its dynamics.

For sUAS flights in France, we observed a positive relationship between sampling time and temperature and concentrations of culturable bacteria. Thus, greater concentrations of bacteria were collected during UAS flights in France that were conducted later in the day and during higher temperatures. During daylight hours, the Earth's surface is heated by the sun, and there is an upward transfer of heat (Oke, 1987). This transfer of heat generates atmospheric turbulence and vertical mixing, stretching the planetary boundary layer kilometer distances (Oke, 1987). We speculate that our observed associations between temperature and sampling time with atmospheric concentrations of bacteria are driven in part by vertical mixing of bacteria from the surface of the Earth. Maldonado-Ramirez et al. (2005) collected viable spores of the fungus *Fusarium graminearum* over 158 UAS sampling flights in New York, USA over four years (Maldonado-Ramirez et al., 2005). Though these authors did not observe a significant difference between spore concentrations among UAS flights conducted during different times of the day, these authors did observe significant differences among UAS flights conducted during clear, cloudy, and rainy conditions. For sUAS flights in Louisiana, we did not observe a significant relationship between temperature and sampling time with concentrations of culturable bacteria (*P* > 0.05 for all variables). Temperatures were much greater during sUAS flights in Louisiana than in France, suggesting potential transport and/or viability limitations during the sampling conditions encountered in Louisiana. Environmental factors such as ultraviolet radiation, relative humidity, and temperature are known to have a significant impact on the viability of microorganisms in the atmosphere and thus may impact their ability to survive transport over long distances (Isard and Gage, 2001; Schmale and Ross, 2015). The identification and tracking of overlapping back-trajectories in high probability regions (Schmale and Ross, 2015) and Lagrangian coherent structures (LCS) (Tallapragada et al., 2011) could help generate hypotheses concerning potential sources and transport distances of the microbes collected during our sUAS missions, but ecological and environmental factors impacting their viability during transit must also be considered.

Atmospheric assemblages of bacteria from sUAS flights in France (TSA) and sUAS flights in Louisiana (R4A) showed more similarity within locations than between locations. Moreover, bacteria collected with sUAS on TSA in France and Virginia were significantly different across all levels of classification tested (*P* < 0.001 for class, order, family, and genus). Cuthbertson et al. (2017) suggested that day of sampling (temporal) is more important than location (spatial) with regards to sample diversity, most likely due to changes in meteorological conditions such as wind direction which appeared to produce distinct assemblages at the level of phylum. Bowers et al. (2009) sampled microbes from the atmosphere at a high-elevation site in Colorado, and reported that microbial abundances, diversity, and composition were fairly stable over a two-week sampling period (Bowers et al., 2009). Another study by Bowers et al. (2011), showed that the composition of the microbes in the sample was linked to the source of the sample (soil, plant leaf, freshwater, etc.) (Bowers et al., 2011). These authors also argued that land-use type was significantly related to the bacterial assemblage composition in the atmosphere, where the most abundant phyla were *Proteobacteria, Actinobacteria* and *Firmicutes*. Interestingly, these three phyla have also been reported from environmental samples collected in Japan (together with *Cyanobacteria* and *Acidobacteria*) (Hu et al., 2017), France (together with *Bacteroidetes*) (Amato et al., 2017), Svalbard (close to the North Pole) (Cuthbertson et al., 2017), and from our sUAS collections reported in this manuscript. Amato et al. (2017) reported diverse microbial assemblages in cloud water samples collected at the Puy de Dôme in France, potentially from a diverse range of sources. Teasing out contributions of local and more distant sources to the sUAS assemblages would require a coordinated sampling effort where multiple sUAS would sample at multiple sites simultaneously. Such an effort is needed to dissect local (the Pujaut, France site was surrounded by farmland, and Louisiana and Virginia sites were on farmland) and more regional sources which could be attributed to larger land-use trends.

Principal Coordinates Analysis showed a strong association between the genera *Curtobacterium, Pantoea*, and *Pseudomonas* from sUAS flights in Virginia, and *Agrococcus, Lysinibacillus*, and *Paenibacillus* from sUAS flights in France. We speculate that these associations are likely due to differences in potential sources and/or weather patterns in these regions (Monteil et al., 2014), but this was not tested in the current study. Such speculation is reasonable given potential linkages between microbial diversity in collections and the attribution of land-use types reported by others (Bowers et al., 2011; Monteil et al., 2014). The weather data we report surrounding our sUAS collections (Table 2) suggest that there may be potential associations between microbial diversity, land-use, and weather, at least for France (where we observed significant relationships between temperature and time of sampling).

A droplet freezing assay was used to screen nearly 2,000 colonies for ice nucleation activity, and 15 colonies were INA at temperatures warmer than −8°C following the initial screen. Only one of these isolates (*Xanthomonas*) was INA following a second screen. Failor et al. (2017) suggested that discrepancies in INA between initial screens of bacteria and repeated screens (following storage and re-plating) could be attributed to growth conditions not conducive to INA. Hanlon et al. (2017) performed INA assays on microbes collected from bulk rain samples from a series of simulated rain events, and found about 1.5% (121/8,331) of the colonies tested following an initial screen (Hanlon et al., 2017). However, only 0.4% (34/8,331) of microbial ice nucleators were INA following a second screen. Powers et al. (2018b) reported about 1% (7/720) of aquatic microbes collected from a freshwater lake in Virginia, USA (Powers et al., 2018b). Likewise, Lindeman and colleagues (Lindemann et al., 1982) showed that on average about 1.7% of the bacteria recovered in air samples above crops were INA and were not greater than 4% of the total viable bacteria detected in the air. On the other hand, about 16% (7/44) of the strains isolated from cloud water on the Puy de Dôme in France were INA (Joly et al., 2013). Failor et al. (2017) isolated ice+ bacteria from bulk rain samples (1.8% of the strains tested, or 593/33,134) from 23 different precipitation events on seven media types, and observed mostly ice+ strains in the class Gammaproteobacteria (Failor et al., 2017). Therefore, the relative abundance of INA bacteria in the total populations that we detected in air in this study were the same order of magnitude as in previous studies of the air, lake water and rainfall, but are an order of magnitude lower than in cloud water (Lindemann et al., 1982; Joly et al., 2013; Failor et al., 2017; Powers et al., 2018b).

Although the quantities of INA bacteria detected here seem very small, when translated into the processes that loft bacteria into the atmosphere and up to cloud heights, these amounts are likely to be very significant relative to cloud processes. Over crops, Lindemann and colleagues (Lindemann et al., 1982) observed net upward bacterial flux of up to 500 bacteria m^−2^ s^−1^ and Carotenuto and colleagues (Carotenuto et al., 2017) observed up to twice that rate for bacteria and fungi combined. Upward flux is sustained by sensible heat flux from sunlight that warms the ground. This usually occurs markedly between about 10:00 to 16:00 on sunny summer days. During this 6-h period, an upward flux of 500 bacteria m^−2^ s^−1^ would contribute a cumulative total of about 10^10^ bacteria into the air over a 1 ha field (and therefore 10^8^ INA bacteria if 1% of these were INA). This suggests that seemingly low concentrations of bacteria in the air (including those observed by sUAS) could nevertheless be of considerable importance as they accumulate over time, and that colossal sampling efforts are needed to capture and quantify them.

Future work aims to understand the potential origin of the atmospheric microbial assemblages collected with sUAS, and their association with mesoscale atmospheric processes. Such work could make use of known sources of ice-nucleating microorganisms in a series of release and recapture experiments (e.g., Prussin et al., 2014), and/or coordinated sampling with sUAS and ground-based collection schemes in periods surrounding major meteorological events such as hurricanes.

## Author contributions

CJ-S cultured microbes, collected and analyzed 16S sequence data, and led the writing of the manuscript. RH conducted ice nucleation assays, managed the strain collection, provided technical advice related to the sequencing, analyzed data, and assisted in writing the manuscript. KA conducted statistical analyses using the 16S data and assisted in writing the manuscript. CP constructed the sUAS sampling devices, assisted in field collections with sUAS in Virginia and Louisiana, and assisted in writing the manuscript. CM helped coordinate field collections in France, analyzed data, and assisted in writing the manuscript. DS managed the project, designed experiments, was the pilot in command (PIC) for all sUAS missions, cultured microbes, analyzed data, and assisted in writing the manuscript.

### Conflict of interest statement

The authors declare that the research was conducted in the absence of any commercial or financial relationships that could be construed as a potential conflict of interest.
